# Knowledge on the Use of Isotretinoin and Its Side Effects and Awareness towards Saudi FDA-Pregnancy Prevention Program among the Female Acne Patients: A Northern Saudi Study

**DOI:** 10.3390/medicina58111609

**Published:** 2022-11-07

**Authors:** Ziad Mansour Alshaalan

**Affiliations:** Division of Dermatology, Department of Internal Medicine, College of Medicine, Jouf University, Sakaka 72388, Saudi Arabia; dr.ziad@ju.edu.sa; Tel.: +966-566000909

**Keywords:** isotretinoin, severe acne, teratogenicity, Saudi FDA-PPP, knowledge, side effects

## Abstract

*Background and Objectives:* Acne vulgaris is one of the most common dermatological disorders among adolescents and adults in the Kingdom of Saudi Arabia (KSA). Isotretinoin is a cost-effective way of treating severe acne patients compared to other methods used for severe forms of acne management. The present study investigated the knowledge of the use of isotretinoin and its side effects among female acne patients of the reproductive age group who were on isotretinoin. This study also assessed participants’ awareness of the Saudi FDA-Pregnancy Prevention Program (SFDA-PPP). *Materials and Methods:* The present population-based cross-sectional survey was conducted among 768 participants using a standard and validated Arabic version questionnaire. We have applied logistic regression analysis to determine the predictors for awareness of SFDA-PPP. A Chi-square test was applied to identify the factors associated with knowledge related to isotretinoin. *Results:* Regarding the side effects of isotretinoin, participated female acne patients were most commonly aware of dry mouth and lips (84.5%), teratogenicity (68.2%), and headache (44.8%). Nearly 60% of the participants belonged to the low knowledge category. The present study participants’ knowledge was significantly associated with education status (*p* = 0.007), occupation (*p* = 0.01), and those participants who were aware of SFDA-PPP (*p* = 0.001). Furthermore, we explored that only 37.5% were aware of the SFDA-PPP program implemented in Saudi Arabia. The awareness of SFDA-PPP was significantly higher among those participants belonging to health sectors (Adjusted OR (95% CI) = 1.39 (1.01–1.92), *p* = 0.049). *Conclusion:* The present survey explored inadequate knowledge among reproductive age group female acne patients regarding isotretinoin uses, precautions to be followed, and side effects, especially teratogenic effects. This survey findings suggest that improving female acne patients’ knowledge of isotretinoin through health promotion activities is crucial, especially by giving them precise instructions about the teratogenic effects.

## 1. Introduction

Acne vulgaris is one of the most common dermatological disorders among adolescents and adults internationally and in the Kingdom of Saudi Arabia (KSA) [1,2]. The prevalence of acne vulgaris during adolescence is higher among males, but during adulthood, it affects females more commonly than males [2,3]. Acne vulgaris can affect the psychosocial life of the affected patients, and this impact is higher among females than males [4,5]. 

Treatment for acne vulgaris ranges from topical retinoids to systemic antibiotics or combined therapy [6]. Isotretinoin is an oral and only effective medication for the cure or prolonged remission of moderate to severe acne, when recommended as monotherapy, improving skin appearance and health-related quality of life [7]. Some authors in the KSA reported that isotretinoin use among females is high. A recent study by Albadr et al. among female college students reported that 48.2% of them used isotretinoin for their acne treatment [8]. Another recent survey from Jeddah city of KSA stated that 22.7% of the females (12–60 years of age group) used isotretinoin at least once for their acne management [9]. The commonly reported adverse effects of the isotretinoin are dry mouth, dry eye, pruritis, photosensitivity, and dysglycemia [7,10]. Even though isotretinoin causes many adverse effects, the teratogenicity caused by this medication is the most serious. It is reported that about one-third of infants exposed to in-utero isotretinoin had the risk of teratogenicity [11,12]. There are several congenital malformations reported by previous researchers, mainly craniofacial defects, cardiovascular and neurological malformations, or thymic disorders [12,13].

In the KSA, the Saudi Food and Drug Authority (SFDA) was established to ensure food and drug safety for humans and animals through different rules and regulations [14]. The below table compares the SFDA-PPP of KSA [14], PPP implemented in the United Kingdom [15], and iPLEDGE from the United States of America [16]. Important features among these programs are compared in Table 1.

The SFDA also specified that oral isotretinoin is subjected to SFDA-Pregnancy Prevention Program (SFDA-PPP). Hence, physicians, pharmacists, and acne patients must be familiar with isotretinoin’s teratogenic effects [17].

Continuous assessment of female acne patients’ awareness regarding isotretinoin uses and its adverse effect, including teratogenicity, is critical for SFDA to plan necessary training and health education sessions for all parties (patients, prescribers, and pharmacists). However, we lack data in the northern region of the KSA. Hence, this study was executed to determine the knowledge of the use of isotretinoin and its side effects among female acne patients of the reproductive age group. This study also assessed awareness of SFDA-PPP and associated factors among them. 

## 2. Materials and Methods

### 2.1. Participants and Setting

The present population-based cross-sectional study was conducted from March 2022 to August 2022 among female acne patients of reproductive age group attending dermatology clinics and outreach clinics conducted at different public places such as malls, masjids, and parks. 

### 2.2. Sample Size

We have concluded the required minimum number of female acne patients using the WHO sample size calculator with the infinite population. To calculate the minimum required sample size, we have taken the confidence interval of 95%, population proportion of 50%, the margin of error of 5%, and the power of the study as 80%. Furthermore, we have taken design effect two. Applying all the values, the estimated sample size was 768 for the current survey. 

### 2.3. Sampling Method

The survey team collected data using a consecutive sampling method. In this method, the data collectors invited the female participants for preliminary screening for the eligibility to be included in the present survey. The study included all acne patients of reproductive age group (aged 18 to 49 years) who were on isotretinoin at the time of data collection and have taken isotretinoin during the past five years (minimum of one treatment course). We excluded the participants who were not willing to participate. The age limit was considered based on the World Health Organization (WHO) classification of the child-bearing age and minimum marriage age limits for girls in the KSA.

### 2.4. Data Collection Procedure

This population-based study questionnaire was distributed to the female acne patients of reproductive age group after ethical clearance from the concerned authorities (Ethics committee, Qurrayat Health Affairs, Qurayyat, Saudi Arabia, Project no: 138, 2022). The data collectors adhered to all COVID-19 prevention strategies instituted by the ministry of health during the study period. Initially, the survey team briefed the study’s objectives to the eligible participants, and informed consent was obtained from them before proceeding with the survey. The acne patients were requested to fill in the google form, the standard and validated tool prepared by the research team based on the existing pieces of literature. The data collection proforma was designed by experts, including a dermatologist, public health specialist, and obstetrician. Hence, we ensured the designed questionnaire represents and covers all facets of the present study’s objectives (face and content validity). We have prepared this proforma from the available works of literature [8,9,18]. The prepared proforma is translated to Arabic by experts in bilingual (English–Arabic). Finally, the Arabic version is back-translated to English by the bilingual non-medical people to that the original meaning is retained. The survey team implemented a pilot study with thirty acne patients on isotretinoin. All patients ensured the constructed questionnaire was clear and easy to understand. The data collection proforma consisted of three parts. Part 1 inquired about the socio-demographic and background details of the acne patients on isotretinoin. The second part inquired about the participants’ knowledge related to the use and side effects of isotretinoin. In the knowledge section, acne patients were given multiple-choice questions to answer. We have given one mark for the correct answer and zero for the wrong answer. After computing all the correct answers of all items, we categorized them into low (≤mean knowledge score) and high (>mean knowledge score). The final part consisted of acne patients’ awareness of the SFDA-PPP program. The research team performed a sub-analysis among the married females and participants who had planned to marry within one month. The present study team assessed the importance of pregnancy tests and contraceptives, as per the SFDA-PPP guidelines received from the care providers.

### 2.5. Statistical Analysis

The completed excel sheet was downloaded, recorded, and analyzed using the statistical package for social sciences (SPSS, Version 24.0). The descriptive statistics of the study are presented as frequency (*n*), proportion (%) for the qualitative variables, and mean and SD for the quantitative variables. We applied the chi-square test to compare the factors associated with the knowledge of isotretinoin and its side effects. Finally, we used binomial logistic regression analysis to find the predictors for the awareness related to SFDA-PPP. All statistical tests applied in this study were two-tailed, and a *p*-value less than 0.05 was fixed as statistically significant. 

## 3. Results

In the present survey, 768 female acne patients participated. Of the participating acne patients, nearly three-fourths (72.3%) studied above high school, the majority (62.9%) of them were single, and 11.0% of the single females had their marriage plans in six months. Among the respondents, 38.8% belong to health sectors, 62.2% live in urban regions, 32.3% had taken isotretinoin during the past year, and 92.7% received isotretinoin with a doctor’s prescription. Regarding SFDA-PPP, only 37.5% were aware of it (Table 2).

Table 3 shows the respondents’ knowledge of use, precautions to be followed while on isotretinoin, and side effects of isotretinoin. Of the responding females, 35.5% were aware of the indication of isotretinoin, 44.9% recognized that patients on isotretinoin must take plenty of water, and only 36.7% answered correctly on blood donation. The most commonly recognized side effects were dry mouth, nose, and lips (84.5%), followed by teratogenicity (68.2%) and headache (44.8%).

After computing all the correct answers of all items, we categorized them into low (≤mean knowledge score) and high (>mean knowledge score). The participants’ knowledge towards isotretinoin was significantly associated with education status (*p* = 0.007), occupation (*p* = 0.01), their current sector (*p* = 0.018), and those participants who were aware of SFDA-PPP (*p* = 0.001) (Table 4).

**Figure 1:**
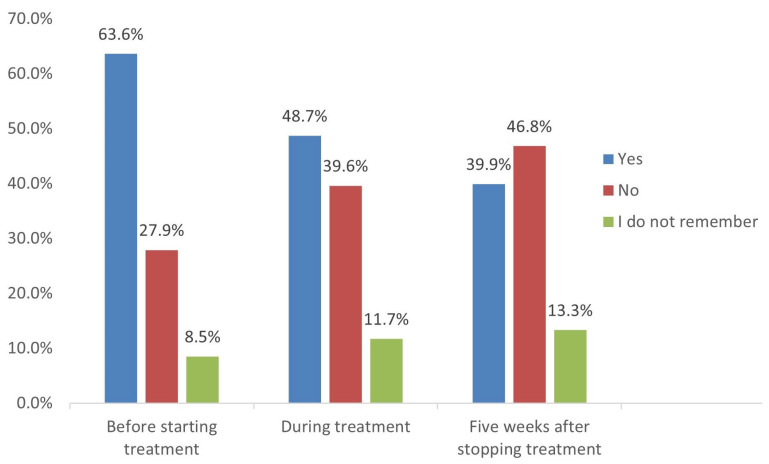
Instructions received about importance of performing pregnancy test (*n* = 308).

**Figure 2:**
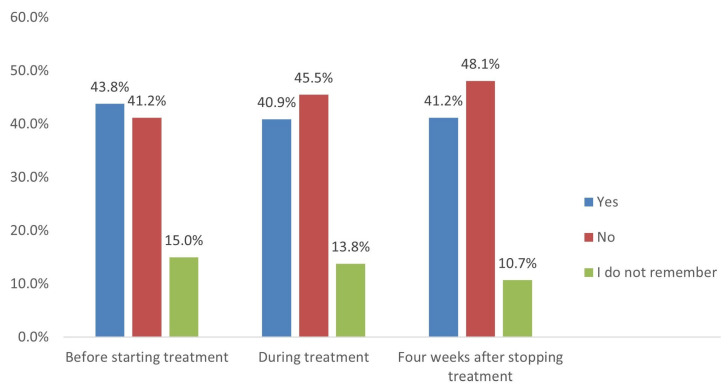
Instructions received about the use of contraceptive methods (*n* = 308).

The research team performed a sub-analysis among the married females and participants who had a plan to marry within one month. Among them (308 participants), we investigated the importance of pregnancy tests and contraceptives, as per the SFDA-PPP guidelines received from the care providers (Figure 1 and Figure 2). Of the 308 participants’ sub-analysis, 63.6% and 48.7% received information on the importance of performing pregnancy tests before and during treatment. In contrast, only 39.9% of them received instructions related to pregnancy tests within five weeks. Regarding the use of contraceptive methods, only less than half of the participants received instructions related to the importance of contraceptive usage before starting treatment (43.8%), during treatment (40.9%), and four weeks after stopping treatment (41.2%).

The present study investigated female acne patients’ awareness of SFDA-PPP. Of the 768 respondents, only 288 (37.5%) were aware of the SFDA-PPP program implemented in the KSA. Using SPSS, the predictors of SFDA-PPP were identified. Firstly, we executed a univariate statistical method (binomial logistic regression) followed by multivariate analysis. The multivariate analysis revealed that awareness of SFDA-PPP was significantly higher among those participants belonging to health sectors (AOR (95% CI) = 1.39 (1.01–1.92), *p* = 0.049), and those who were on isotretinoin in past one year (AOR (95% CI) = 1.55 (1.13–2.14), *p* = 0.007). In contrast, awareness on SFDA-PPP were significantly lower among unemployed participants (AOR (95% CI) = 0.65 (0.41–0.97), *p* = 0.029) and those who work in private sectors (AOR (95% CI) = 0.53 (0.31–0.67), *p* = 0.026) (Table 5 and Table 6).

## 4. Discussion

Isotretinoin is a cost-effective way of treating severe acne patients compared to other methods used for severe forms of acne management [19]. However, it has been proven to cause some severe, including teratogenic, side effects and consumers must be aware of those side effects [20,21]. Hence, the present survey, the first in the northern region, assessed knowledge of the use of isotretinoin and its side effects among female acne patients of the reproductive age group. 

The present study explored that 92.7% of acne patients obtained isotretinoin with a prescription from the doctor. Even though it is expected that isotretinoin must be dispensed with proper prescription and consent forms, our results align with some recently published studies from the KSA [8,9,22]. This may be related to more than just pharmacist dispense, and this could be due to using isotretinoin prescribed for the siblings, purchased while on travel to other countries, etc. These epidemiological survey findings indicate that the knowledge of the community pharmacist must be improved to adhere to policy regarding isotretinoin and the need for SFDA-PPP awareness-raising campaigns beyond consumers (acne patients who is on isotretinoin) [23]. 

The current survey’s female participants recognized dry mouth, nose, and lips (84.5%) as the most common side effects of isotretinoin. Our findings are consistent with previous studies conducted in the KSA and other parts of the world [24,25,26]. Teratogenicity is one of the severe adverse effects of isotretinoin that female acne patients at reproductive age must be aware of. Nonetheless, our survey found that nearly two-thirds (68.2%) were aware of the teratogenic side effects. The low level of awareness regarding this severe side effect is an alarming phenomenon noted in our survey. These results contrast with another study by Younis NS et al. They reported that a higher proportion (88.9%) of the community participants were aware that teratogenicity is a dangerous side effect associated with isotretinoin use [26]. The possible reasons for this dissimilarity could be the study setting, inclusions, and exclusion of the study participants. Our study included female acne patients of the reproductive age group from the northern KSA. Younis NS et al. assessed public awareness towards isotretinoin from the Eastern province of KSA. Furthermore, these differences across the KSA also support the rationale of our study on the necessity of having region-specific data. 

Another interesting finding explored by the present study was related to blood donation. The current population-based survey found that only 63.3% of the respondents knew that patients on isotretinoin should not donate blood during treatment or within 30 days of stopping isotretinoin. A recently published study from Jordan by Jarab AS et al. in 2022 reported a higher proportion of participants aware of not donating blood while on isotretinoin [27]. Another study conducted by Imam SA et al. in 2021 showed that almost half (50.5%) of their study participants did not know that they should not donate blood during the treatment [28].

Patients’ knowledge about the prescribed drugs, risks, benefits, and precautions to follow during treatment is a critical predictor of treatment outcome [29,30]. Our study showed that the participants’ knowledge of isotretinoin uses, and side effects was significantly associated with education status (*p* = 0.007), occupation (*p* = 0.01), and their current working sector (*p* = 0.018). In contrast to our study statement, a study conducted by Jarab AS et al. showed a non-significant association between knowledge of isotretinoin with education and occupation [27]. These contrasting results could be due to respondents’ background characteristics. We have included female acne patients of the reproductive age group, while Jarab AS et al. included both genders in their study. Another published study by Alharbi et al. presented a positive association between gender and some of the side effects of isotretinoin [31].

Some authors in the past explored the awareness of important health-related organizations of the KSA and their activities, including SFDA-PPP [17,32]. However, to the best of our knowledge, the present study is the first one that evaluated the factors associated with awareness of SFDA-PPP. Regarding SFDA-PPP guidelines, the present study found that nearly two-thirds of the married participants and patients who had a plan to marry within a month received information on the importance of performing pregnancy tests before and during treatment. Currently, SFDA-PPP program activities are targeted only at consumers. This could be a possible reason for the present study’s low awareness (37.5%). Interestingly much lower awareness of SFDA-PPP (30.6%) was found by a study in the capital city (Riyadh) of Saudi Arabia [17]. Our study findings are supported by another study by Ibrahim et al. that evaluated SFDA-PPP guidelines received among female acne patients [17].

### Strengths and Limitations

Our study is the first study that assessed awareness of SFDA-PPP and its predictive factors, and certain patients’ characteristics lead to a lesser understanding of risks in northern region. Secondly, all the participants were informed about the uses, side effects (including teratogenicity), and SFDA-PPP activities to all respondents after completing the survey. However, some of the study’s limitations must be considered while reading the present research findings. Firstly, the present population-based study utilized a cross-sectional study design. Hence, we have explored only the association, not the causation. Secondly, this research invited participants through a consecutive sampling method, so the possibility of self-selection bias cannot be excluded. Finally, the present study was conducted in the northern region of KSA. Considering the wide range of socio-cultural variations in the KSA, we cannot apply the results to other regions of the KSA.

## 5. Conclusions

The present survey explored inadequate knowledge among reproductive age group female acne patients regarding isotretinoin uses, precautions to be followed, and side effects, especially teratogenic effects. Hence, improving female acne patients’ knowledge through health promotion activities is crucial, especially precise instruction about the teratogenic effects of isotretinoin must be given. We found a low-level awareness among the participants towards SFDA-PPP activities. Furthermore, we revealed that nearly one-third of the married participants did not receive SFDA-PPP guidelines on pregnancy tests and contraception methods to be followed. Therefore, awareness-raising campaigns about SFDA-PPP and its activities are to be implemented by the responsible authorities. These campaigns should be targeted to all females of reproductive age group, including acne patients. Finally, the present study aimed to understand the patients’ knowledge and certain patient’s characteristics lead to lesser understanding of risks. The present study’s findings indicate a need for future research that to be conducted among other two stakeholders (pharmacists and doctors) of SFDA-PPP.

## Figures and Tables

**Table 1:** Pregnancy prevention programs comparison among KSA, USA, and UK.

Salient Features	SFDA-PPP (KSA)	iPLEDGE (USA)	PPP (UK)
Year implemented	2013	2006	1988
Prescribers	Prescribed by experts (dermatologists and family physicians) registered with Saudi commission for health specialties	Only iPLEDGE enrolled and activated healthcare providers can prescribe isotretinoin.	Can only be prescribed within a team led by a consultant dermatologist
Contraception advice	The physician should ensure that the patient has two effective contraception methods; importantly, they should include a barrier device as a second method.	The physician should ensure that the patient has two methods effective contraception methods. Importantly they should include a barrier device as a second method.	Contraception should commence 1 month before treatment and progress until the patient is free from retinoid.
Pregnancy test	Treatment is subject to pregnancy tests.	A pregnancy test must be taken before a patient is subjected to oral isotretinoin.	All pregnancy tests that fall under 25 mlU/mL sensitivity should be recorded 3 days before the prescription.
Prescription duration from a single visit	Limited to 4 weeks. Monthly renewal (if required).	Limited to 4 weeks. Monthly renewal (if required).	Limited to 4 weeks. Monthly renewal (if required)
In the event pregnancy happened	Treatment is stopped immediately, and the patient should be referred to a specialist in teratogenicity for advice.	Treatment must be stopped immediately if the woman being treated with oral Isotretinoin is pregnant.	Treatment should be excluded for pregnant women.
Distribution system	A distribution control system is established to limit the misuse.	Available only through a restricted distribution program.	A restricted distribution is established and monitored. Isotretinoin to be given within 7 days of issuance of prescription.

**Table 2:** Background characteristics of the acne patients who participated in the study (*n* = 768).

Background Characteristics	Frequency	Percentage
Age: Mean (SD)	30.17 (7.52)	
≤30 Years	377	49.1
>30 Years	391	50.9
Education status		
Up to high school	213	27.7
Above high school	555	72.3
Marital status		
Single	483	62.9
Married	251	32.7
Divorced/Widowed	34	4.4
Marriage plan within one month *		
No	371	71.7
Yes	57	11
Not sure	89	17.2
Occupation		
Government	208	21
Private sector/business	130	16.9
Unemployed	161	27.1
Student	269	35
Occupation/Education sector		
Non-Health	470	61.2
Health	298	38.8
Living place		
Urban	478	62.2
Rural	290	37.8
Isotretinoin use		
Past one year	248	32.3
Before one year	520	67.7
Have you taken isotretinoin with a doctor’s prescription?		
Yes	712	92.7
No	56	7.3
Awareness of SFDA-PPP		
Yes	288	37.5
No	480	62.5

* Not applicable for married participants.

**Table 3:** Participants’ knowledge of the use, precautions, and side effects of isotretinoin (*n* = 768).

Items	Correct Answer *n* (%)	Wrong Answer *n* (%)
Type of acne patients prescribed with isotretinoin	273 (35.5)	495 (64.5)
Patients who are on isotretinoin must take plenty of water	345 (44.9)	423 (55.1)
Maximum daily dosage of isotretinoin	379 (49.3)	389 (50.7)
Patients can donate blood while on isotretinoin treatment	486 (63.3)	282 (36.7)
Patients can take isotretinoin for more than six months without stopping	561 (73.0)	207 (27.0)
Side effects—Dry mouth, nose, and lips	649 (84.5)	119 (15.5)
Side effects—Skin rashes	300 (39.1)	468 (60.9)
Side effects—Headache	344 (44.8)	424 (55.2)
Side effects—Dysglycemia	196 (25.5)	572 (74.5)
Side effects—Teratogenicity	524 (68.2)	244 (31.8)
Mean ± SD of the total score	5.03 ± 1.76	

**Table 4:** Association between background characteristics and knowledge category (Statistical test applied—Chi-Square test).

Background Characteristics	Low	High	*p*-Value
	*n* (%)	*n* (%)	
Age			
≤30 Years	240 (63.7)	137 (36.3)	0.074
>30 Years	228 (58.3)	163 (41.7)	
Education status			
Up to high school	146 (68.5)	67(31.5)	0.007 *
Above high school	322 (58.0)	233 (42.0)	
Marital status			
Single	285 (59.0)	198 (41.0)	0.328
Married	160 (63.7)	91 (36.3)	
Divorced/Widowed	23 (67.6)	11 (32.4)	
Occupation			
Government	111 (53.4)	97 (46.6)	0.010 *
Private sector/business	71 (54.6)	59 (45.4)	
Unemployed	113 (70.2)	48 (29.8)	
Student	166 (61.7)	103 (38.3)	
Occupation/Education sector			
Non-Health	302 (64.3)	168 (35.7)	0.018 *
Health	166 (55.7)	132 (44.3)	
Living place			
Urban	287 (60.0)	191 (40.0)	0.513
Rural	181 (62.4)	109 (37.6)	
Isotretinoin use			
Past one year	327 (62.9)	193 (37.1)	0.109
Before one year	141 (56.9)	107 (43.1)	
Awareness SFDA-PPP			
Yes	330 (68.8)	150 (31.3)	0.001 *
No	138 (47.9)	150 (52.1)	

* Significant value at *p* value <0.05

**Table 5:** Factors associated with the awareness of SFDA-PPP: Univariate analysis (Statistical test applied—binomial logistic regression).

Background Characteristics	Total	Saudi FDA Awareness			
		No (480)	Yes (280)	Unadjusted Odds	*p*-Value **
				Ratio (95% CI of OR) *	
Age					
≤30 Years	377	231	146	Ref.	
>30 Years	391	249	142	0.90 (0.67–1.21)	0.491
Education status					
Up to high school	213	142	71	Ref.	
Above high school	555	338	217	1.28 (0.92–1.79)	0.14
Marital status					
Single	517	314	203	Ref.	
Married	251	166	85	0.79 (0.58–1.09)	0.147
Occupation					
Government	208	119	89	Ref.	
Private sector/business	130	88	42	1.57 (0.99–2.48)	0.054
Unemployed	161	105	56	1.40 (0.92–2.15)	0.118
Student	269	168	101	1.24 (0.86–1.80)	0.247
Occupation/Education sector					
Non-Health	470	307	163	Ref.	
Health	298	173	125	1.36 (1.01–1.83)	0.043 **
Living place					
Urban	478	181	297	Ref.	
Rural	290	107	183	0.96 (0.79–1.28)	0.817
Isotretinoin use					
Past one year	248	139	109	Ref.	
Before one year	520	341	179	1.49 (1.08–2.04)	0.011 **

* Binomial logistic regression (enter method): Univariate and unadjusted OR. ** Significant value at 0.05.

**Table 6:** Factors associated with the awareness about Saudi FDA—Multivariate analysis (Statistical test applied—binomial logistic regression).

Background Characteristics	Total	Saudi FDA Awareness			
		No (480)	Yes (288)	Adjusted Odds Ratio (95% CI of AOR) *	*p*-Value **
Age					
≤30 Years	377	231	146	Ref.	
>30 Years	391	249	142	0.76 (0.49–1.25)	0.308
Education status					
Up to high school	213	142	71	Ref.	
Above high school	555	338	217	1.26 (0.88–1.79)	0.201
Marital status					
Single	517	314	203	Ref.	
Married	251	166	85	0.79 (0.53–1.17)	0.236
Occupation					
Government	208	119	89	Ref.	
Private sector/business	130	88	42	0.53 (0.31–0.67)	0.026 **
Unemployed	161	105	56	0.65 (0.41–0.97)	0.029 **
Student	269	168	101	0.72 (0.45–1.15)	0.173
Occupation/Education sector					
Non-Health	470	307	163	Ref.	
Health	298	173	125	1.39 (1.01–1.92)	0.049 **
Living place					
Urban	478	181	297	Ref.	
Rural	290	107	183	0.94 (0.59–1.48)	0.792
Isotretinoin use					
Before one year	520	341	179	Ref.	
Past one year	248	139	109	1.55 (1.13–2.14)	0.007 **

* Binomial logistic regression (enter method): Adjusted variables age, education status, marital status, occupation, current education/occupation section, living place, and isotretinoin use. ** Significant value at 0.05.

## Data Availability

The data presented in this study are available on request from the corresponding author.
